# Supplementary figures and images for: Deep immunophenotyping reveals endometriosis is marked by dysregulation of the mononuclear phagocytic system in endometrium and peripheral blood
Source: BMC Med. 2022 Apr 15;20:158. doi: 10.1186/s12916-022-02359-4 (PMC9011995; doi:10.1186/s12916-022-02359-4)

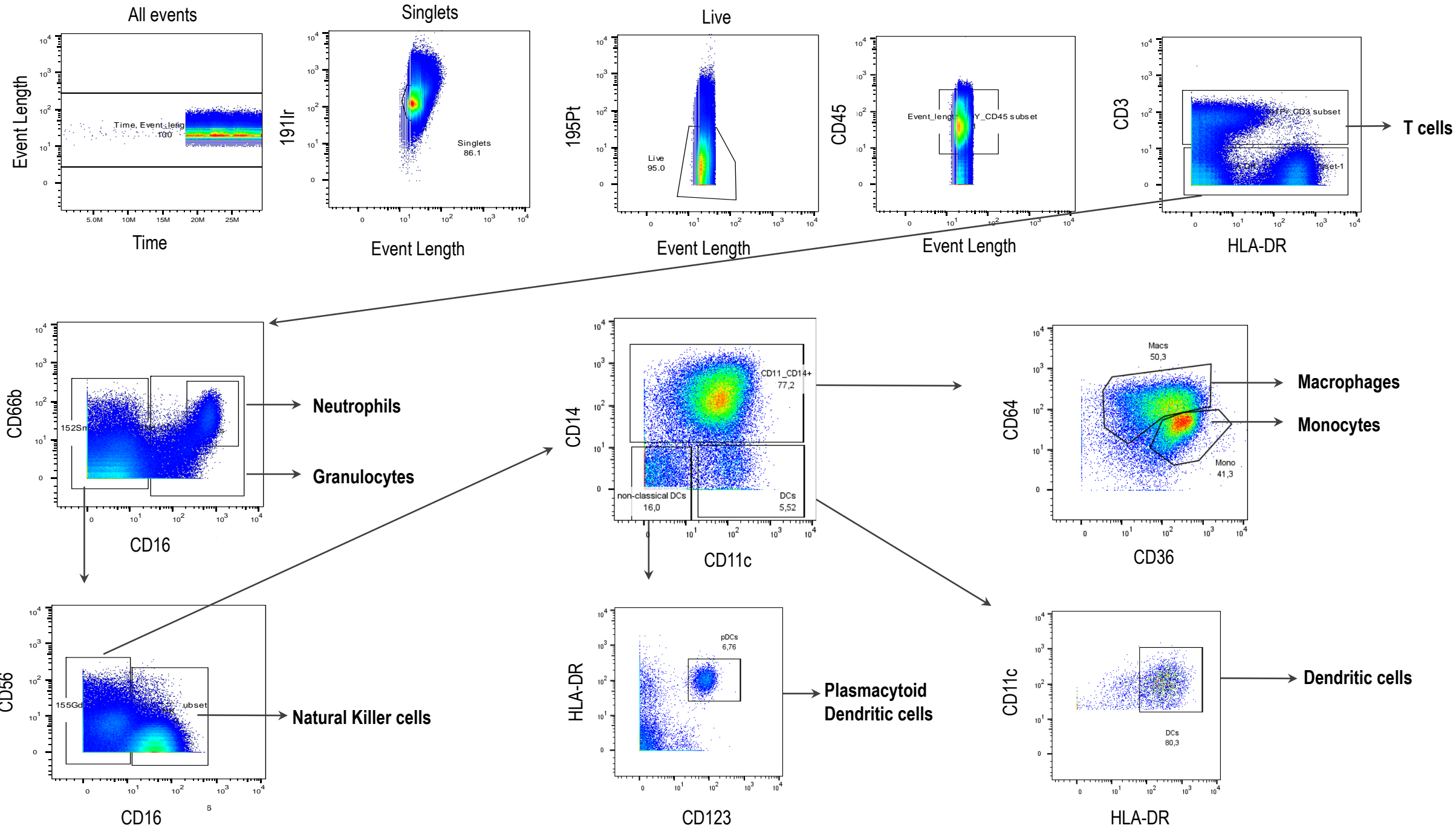

Supplement: Supplementary file 3 — Additional file 3: Fig. S1. Manual gating strategy for the focused panel. The figure shows an example of the dot plots obtained using FlowJo in endometrial cells. [file 12916_2022_2359_MOESM3_ESM.pdf]

A.

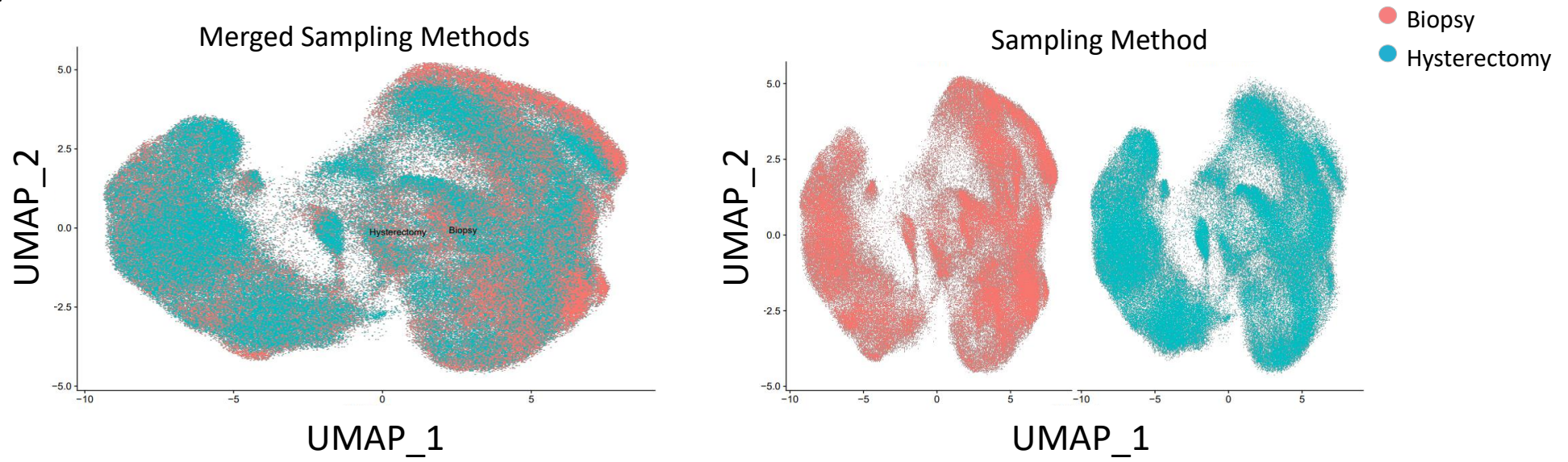

B.

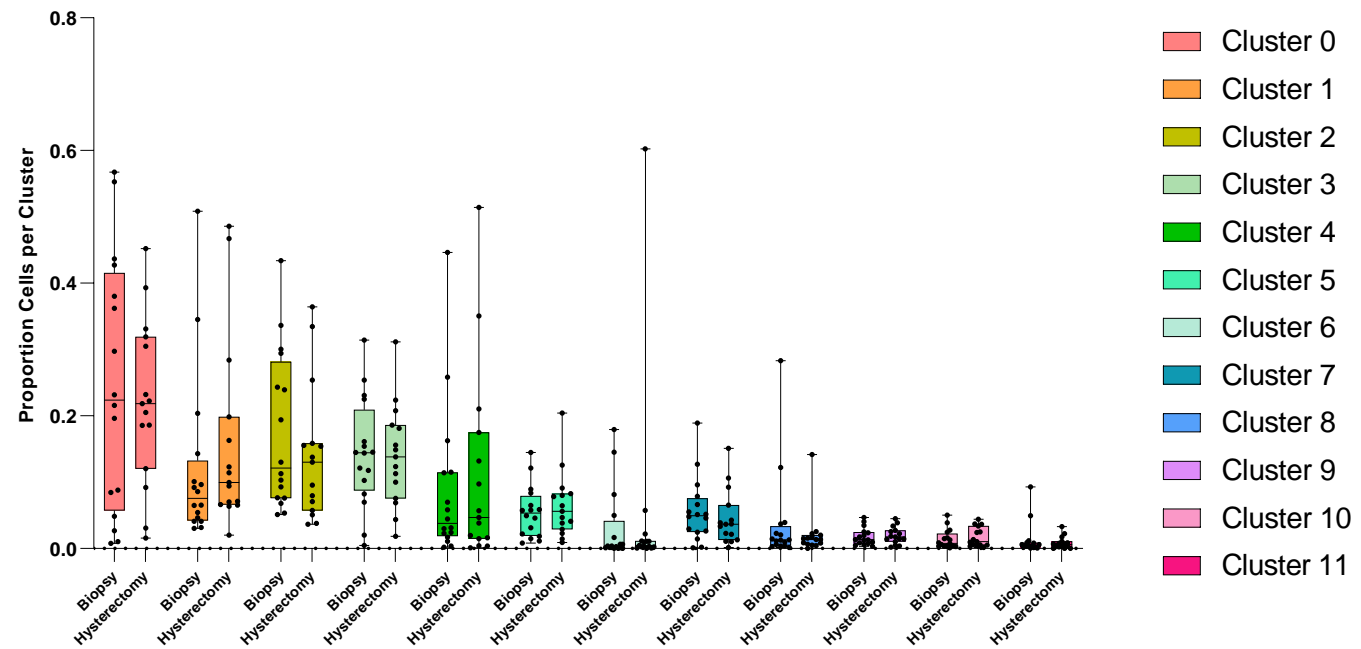

Supplement: Supplementary file 4 — Additional file 4: Fig. S2. Endometrial sampling methods. A) The figure shows UMAPs of the two methods used for endometrial tissue collection, biopsy (red) and hysterectomy (blue). The left panel shows a UMAP with the overlap between the two techniques and the right panel the split UMAPs for each technique. n=17 endometrial biopsies and n=17 hysterectomies. B) The graph shows the proportion of cells in each cluster obtained by the different sampling methods. [file 12916_2022_2359_MOESM4_ESM.pdf]

Endometrium

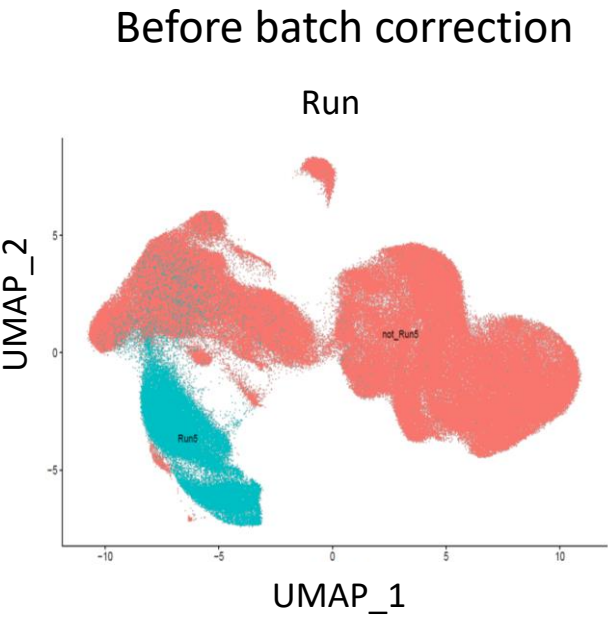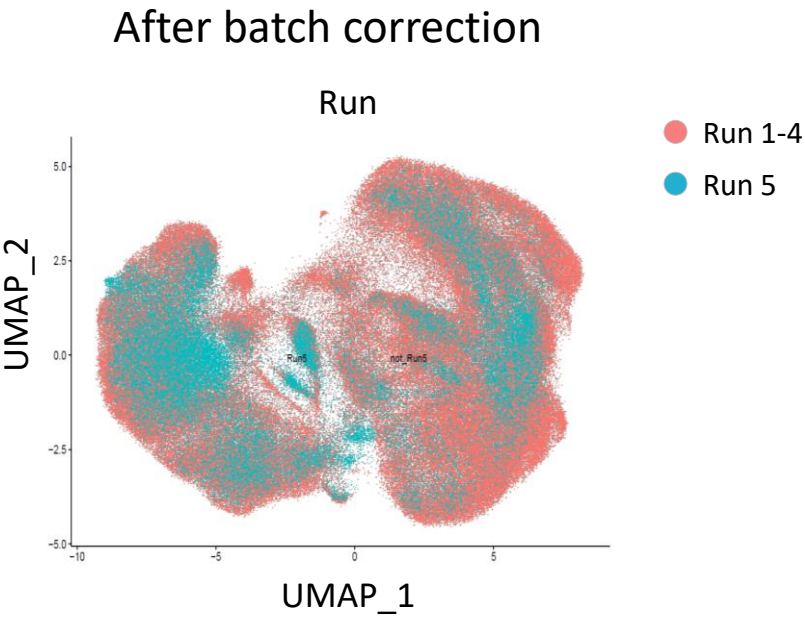

PBMCs

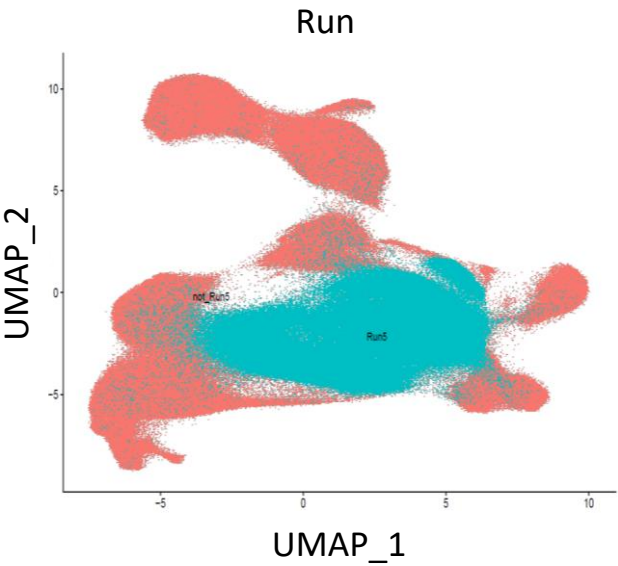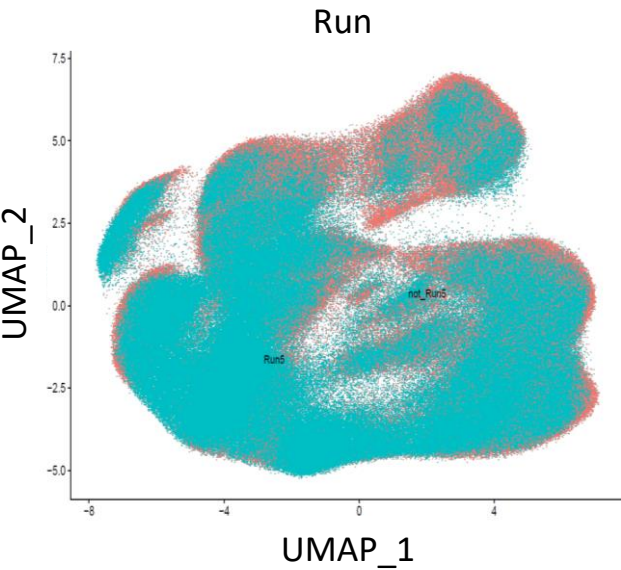

Supplement: Supplementary file 5 — Additional file 5: Fig. S3. Batch effects derived from different runs. The figure shows UMAPs representing the distribution of the cells from the runs in the CyTOF instrument for both endometrium (top panel) and PBMCs (bottom panel). The figure shows the UMAPs before (left panel) and after (right panel) batch correction. Red: runs 1 to 4; blue: run 5. [file 12916_2022_2359_MOESM5_ESM.pdf]

A.

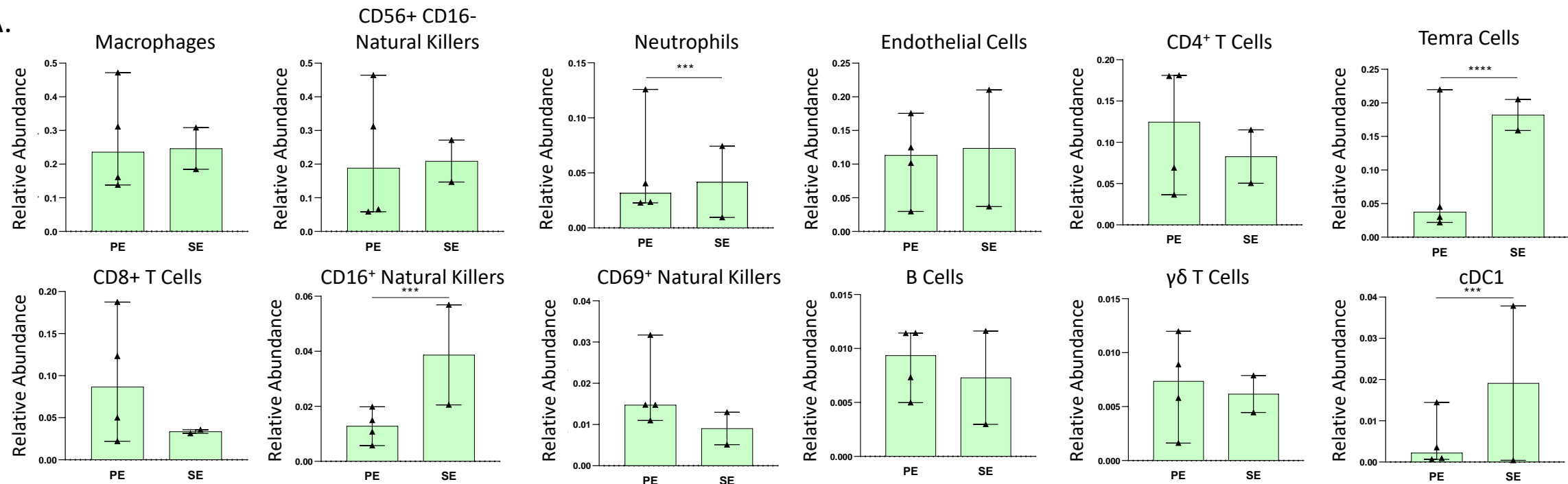

B.

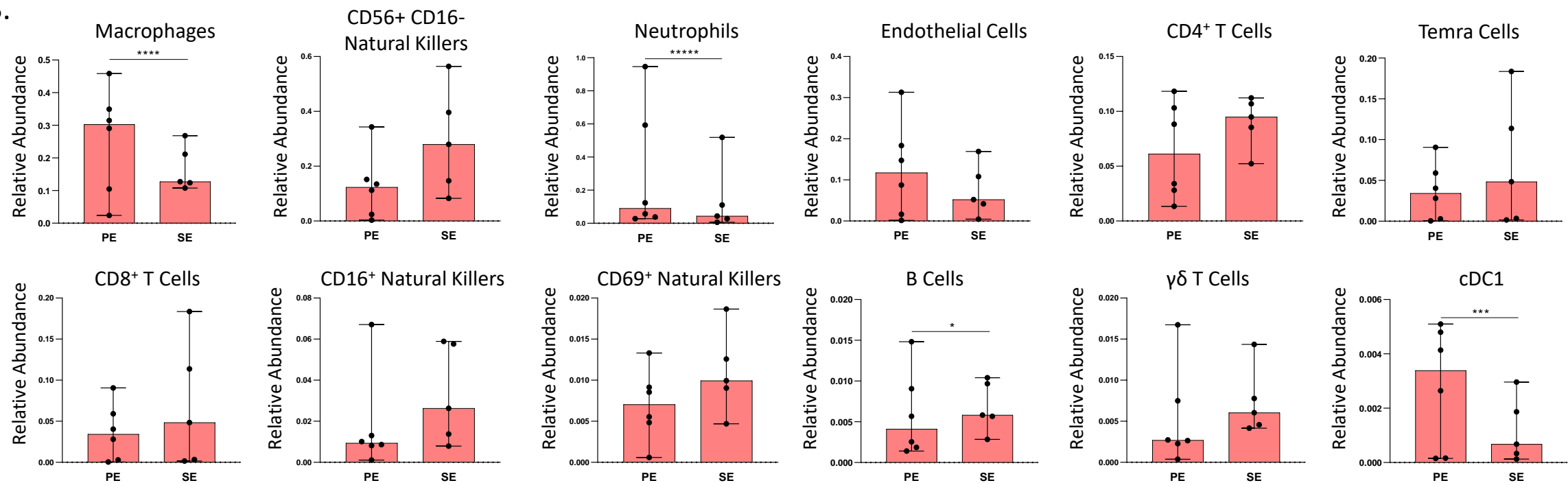

Supplement: Supplementary file 7 — Additional file 7: Fig. S5. Fluctuation of all endometrial immune populations identified in the broad panel. A) Differences in abundance of populations throughout the menstrual cycle in control samples. B) Differences in abundance of populations throughout the menstrual cycle in samples from women with endometriosis. pValues: *, pVal ≤ 0.05; **, pVal ≤ 0.005; ***, pVal ≤ 0.0005; **** pVal ≤ 0.00005. n=17 (4 controls PE, 2 controls SE, 6 endometriosis PE, 5 endometriosis SE). PE: proliferative, SE: secretory. [file 12916_2022_2359_MOESM7_ESM.pdf]

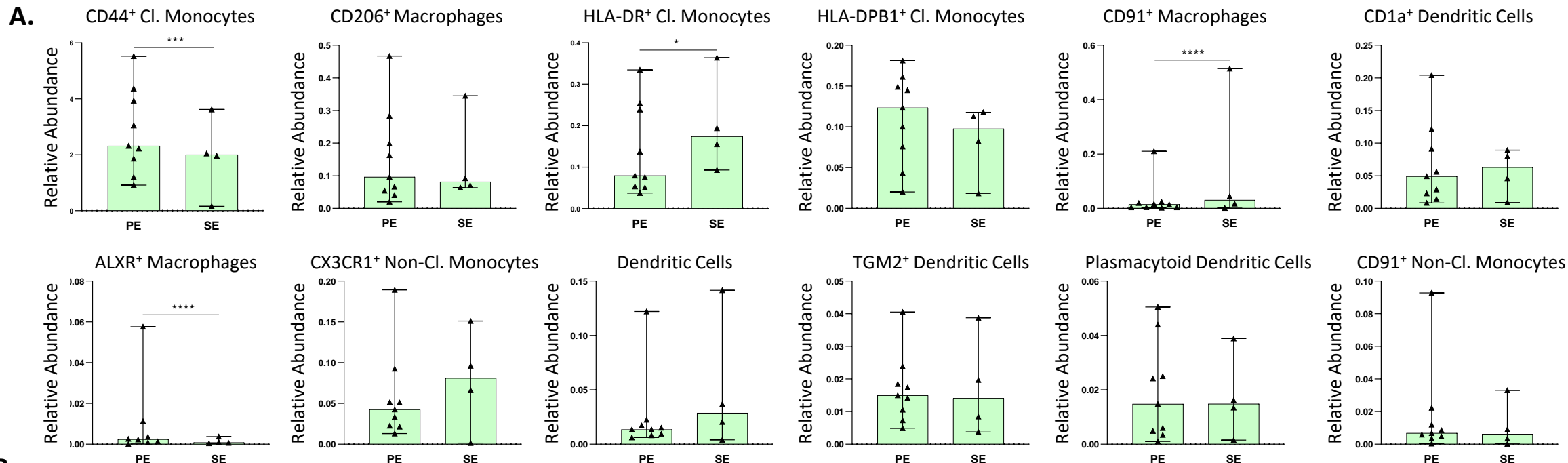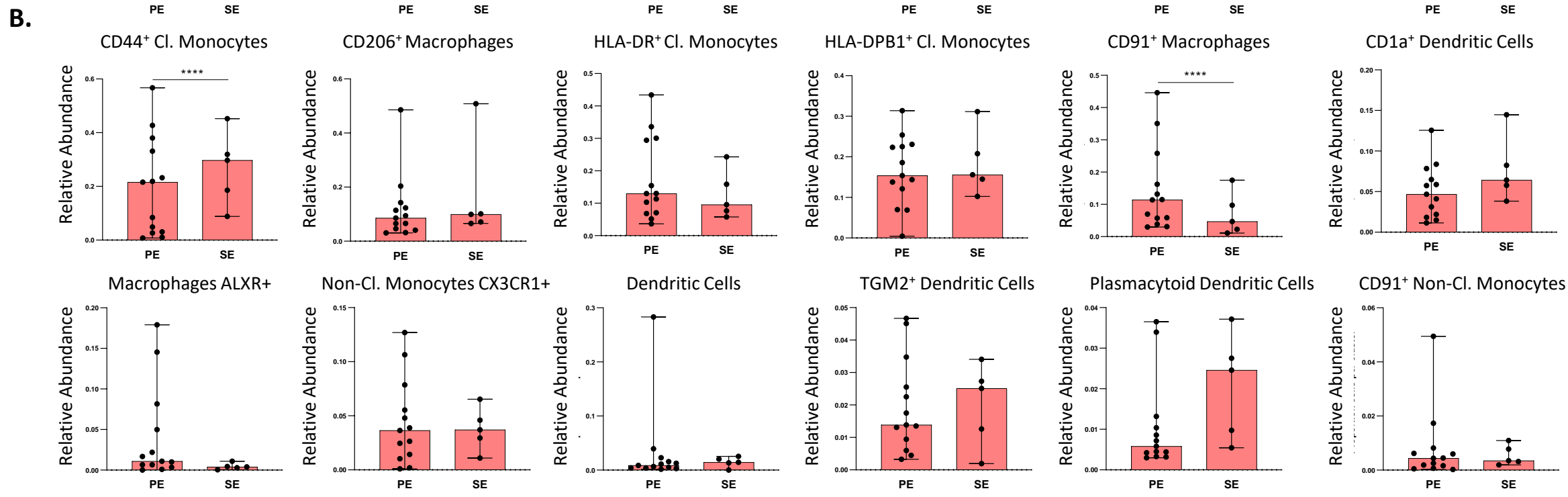

Supplement: Supplementary file 8 — Additional file 8: Fig. S6. Fluctuation of all endometrial immune populations identified in the focused panel. A) Differences in abundance of populations throughout the menstrual cycle in control samples. B) Differences in abundance of populations throughout the menstrual cycle in samples from women with endometriosis. pValues: *, pVal ≤ 0.05; **, pVal ≤ 0.005; ***, pVal ≤ 0.0005; **** pVal ≤ 0.00005. n=13 controls (9 PE and 4 SE) and n=18 endometriosis (13 PE (8 mild and 5 severe stages) and 5 SE (all mild stage of disease)). (PE: proliferative, SE: secretory). [file 12916_2022_2359_MOESM8_ESM.pdf]
