# Supplementary material for: Deep immunophenotyping reveals endometriosis is marked by dysregulation of the mononuclear phagocytic system in endometrium and peripheral blood
Source: BMC Med. 2022 Apr 15;20:158. doi: 10.1186/s12916-022-02359-4 (PMC9011995; doi:10.1186/s12916-022-02359-4)
Supplement: Supplementary file 6 — Additional file 6: Fig. S4. Proportion of endometrial immune populations identified by using the broad panel. The figure shows the proportion of each population in the ring graphs and the percentage of each population is also shown in each condition of study. Ctrl_PE (n=4), Ctrl_SE (n=2), Endo_PE (n=6) Endo_SE (n=5). Ctrl: control, Endo: endometriosis, PE: proliferative, SE: secretory. [file 12916_2022_2359_MOESM6_ESM.pdf]

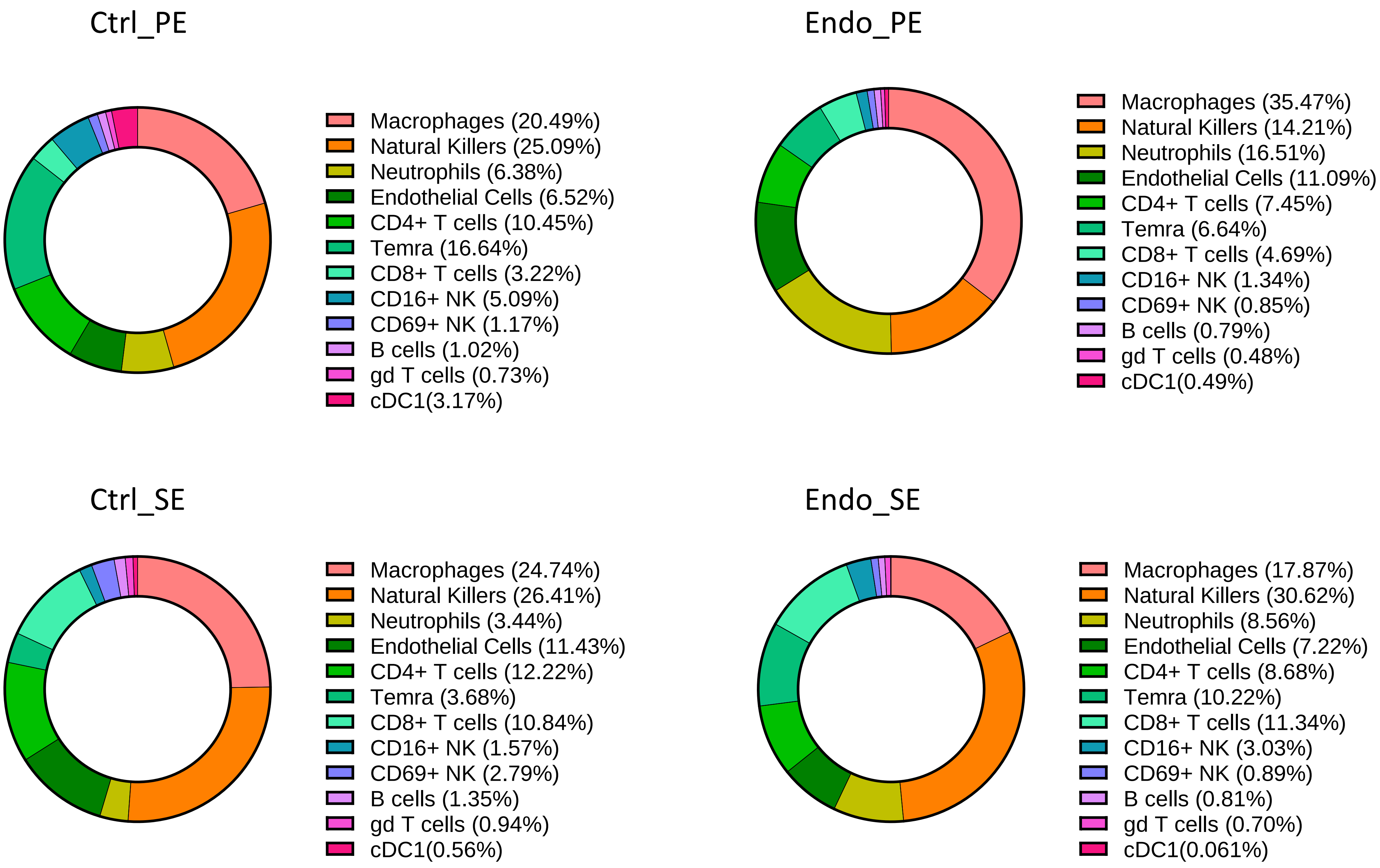
Ctrl_PE
Macrophages (20.49%)
Natural Killers (25.09%)
Neutrophils (6.38%)
Endothelial Cells (6.52%)
CD4+ T cells (10.45%)
Temra (16.64%)
CD8+ T cells (3.22%)
CD16+ NK (5.09%)
CD69+ NK (1.17%)
B cells (1.02%)
gd T cells (0.73%)
cDC1(3.17%)
Endo_PE
Macrophages (35.47%)
Natural Killers (14.21%)
Neutrophils (16.51%)
Endothelial Cells (11.09%)
CD4+ T cells (7.45%)
Temra (6.64%)
CD8+ T cells (4.69%)
CD16+ NK (1.34%)
CD69+ NK (0.85%)
B cells (0.79%)
gd T cells (0.48%)
cDC1(0.49%)
Ctrl_SE
Macrophages (24.74%)
Natural Killers (26.41%)
Neutrophils (3.44%)
Endothelial Cells (11.43%)
CD4+ T cells (12.22%)
Temra (3.68%)
CD8+ T cells (10.84%)
CD16+ NK (1.57%)
CD69+ NK (2.79%)
B cells (1.35%)
gd T cells (0.94%)
cDC1(0.56%)
Endo_SE
Macrophages (17.87%)
Natural Killers (30.62%)
Neutrophils (8.56%)
Endothelial Cells (7.22%)
CD4+ T cells (8.68%)
Temra (10.22%)
CD8+ T cells (11.34%)
CD16+ NK (3.03%)
CD69+ NK (0.89%)
B cells (0.81%)
gd T cells (0.70%)
cDC1(0.061%)
